# Advancements in Drug Delivery Systems in Glioblastoma Therapy

**DOI:** 10.3390/ijms27052298

**Published:** 2026-02-28

**Authors:** Purusottam Mishra, Payal Gupta, Aleksandra Markowska, Saeid Ghavami, Jarosław Markowski, Marek J. Łos

**Affiliations:** 1 Biotechnology Center, Silesian University of Technology, 44-100 Gliwice, Poland; pmishra@polsl.pl; 2 Department of Biotechnology, Graphic Era (Deemed to be University), Dehradun 248002, India; payalgupta.bt@geu.ac.in; 3 Faculty of Medicine, Medical University of Warsaw, 02-091 Warsaw, Poland; 4 Department of Human Anatomy and Cell Science, Rady Faculty of Health Sciences, Max Rady College of Medicine, University of Manitoba, Winnipeg, MB R3E 0T6, Canada; saeid.ghavami@gmail.com; 5 Paul Albrechtsen Research Institute, Cancer Care Manitoba, University of Manitoba, Winnipeg, MB R3E 0V9, Canada; 6 Children Hospital Research Institute of Manitoba, University of Manitoba, Winnipeg, MB R3E 3P4, Canada; 7 Faculty of Medicine, Akademia Śląska, Ul Rolna, 43, 40-555 Katowice, Poland; 8 Department of Laryngology, Faculty of Medical Sciences in Katowice, Medical University of Silesia, 40-027 Katowice, Poland

**Keywords:** glioblastoma, drug delivery, blood-brain barrier, tumour and cancer

## Abstract

Glioblastoma (GB) is one of the most aggressive brain tumours, with a high mortality rate. Tumour heterogeneity, GB’s invasive nature, the blood–brain barrier (BBB) and resistance development offer significant challenges in devising an effective strategy to manage GB. Clinicians rely on tumour resection, radiotherapy and temozolomide (TMZ) chemotherapy, but their efficacy is hindered due to poor BBB penetration. EGFR (epidermal growth factor receptor), NF-κB, angiogenic pathways, RAS/RAF/MAPK, PI3K/Akt/mTOR, etc., play an important role in GB progression. Development in nanotechnology, pharmaceutical science and genetic engineering enables the design of drug candidates with superior efficacy and safety profiles. This review delves into recent advancements in nanoparticles, hydrogels, extracellular vesicles, microneedles and other drug delivery platforms used in GB treatment. These novel drug delivery systems achieved superior BBB penetration, tumour targeting, and controlled release and better survival outcomes in preclinical setups. This review also discusses the major translational challenges, including those of large-scale production, tumour heterogeneity, off-target effects and M2 macrophage induction. Innovative strategies focusing on drug delivery as a biological decision-making process, integrating tumour stress responses into drug carrier and system-level design principles, are discussed, outlining future prospects.

## 1. Introduction

Glioblastoma (GB) is a highly malignant subtype of brain tumour caused by the unregulated growth of glial cells [1,2]. It commonly impacts the frontal and temporal lobes of the brain, but it may also impact other parts of the brain. Around 4 in 100,000 adults are diagnosed with GB, whereas the incidence rate in the population under 18 years is significantly lower [3]. GB is most prevalent in the population aged between 65 and 84 years [4]. Due to its extremely complex characteristics and high malignancy, the survival rate of GB is less than 7% and the mean survival time is around 8 months [5,6]. Common symptoms of GB are fatigue, cognitive impairment, confusion, headaches, motor impairments, breathlessness, seizures, drowsiness and language difficulties [4,7]. Molecular pathways such as EGFR (epidermal growth factor receptor), NF-κB, angiogenic pathways, RAS/RAF/MAPK, PI3K/Akt/mTOR, Notch, Wnt, and Hedgehog were found to play a key role in GB progression [8,9,10]. On the other hand, m6A RNA methylation, m7G RNA methylation, m5C RNA modifications and chaperone-mediated autophagy also enhance the chances of GB occurrence [11,12,13]. These highly complex interlinked biological pathways make GB treatment difficult and lower the survival chances.

Physicians use surgical procedures for tumour resection with radiation therapy, chemotherapy and TMZ to manage GB, but its recurrence is inevitable. However, interdisciplinary research involving engineers, doctors and biochemists has made significant progress in the areas of immunotherapy, thermotherapy, novel drug delivery systems, antiangiogenic approaches, gene therapy, etc. However, the presence of the blood–brain barrier (BBB), blood–tumour barrier (BTB), tumour complexity, and cancer stem cells are major challenges that need to be addressed to enhance the therapeutic outcomes of these new strategies [14,15,16].

The US Food and Drug Administration (FDA) has approved five drugs for treating GB: lomustine, carmustine, bevacizumab, TMZ, and vorasidenib, as these drug molecules are capable of crossing the BBB [17,18,19]. Previously published results showed that lomustine, carmustine, TMZ and vorasidenib cross the BBB due to their lipophilic structure and passive diffusion [19,20,21,22]. However, bevacizumab preferably penetrates the leaky BBB in higher-grade GB [23]. In vitro studies showed excellent cytotoxic properties of paclitaxel and doxorubicin (Dox) in the presence of GB cells, but in clinical settings, poor BBB penetration reduces their therapeutic outcome in GB patients [24,25,26,27]. Both Dox and paclitaxel serve as substrates to multidrug resistance (MDR) efflux transporters. As a result, both drug molecules are rapidly eliminated from the brain tissue [28,29,30].

Advancements in nanotechnology and biomedical and interdisciplinary research have enhanced the therapeutic potency of GB-targeting drug molecules [31,32]. Next-generation drug delivery platforms enable the selective release of therapeutic agents in the tumour environment with minimal side effects. Therefore, novel drug delivery systems could be a game-changer in treating GB in the near future.

In this review, we have highlighted the molecular mechanisms leading to GB and the factors responsible for lowering the sensitivity of the drug molecules against it. New therapeutic interventions, such as brain-targeted drug delivery systems, nanoparticles, hydrogels, liposomes, micelles, wafer implants, catheter implants, dendrimers, etc., will be discussed. The existing challenges alongside future prospects are also outlined in this review.

## 2. Molecular Mechanisms Underlying Glioblastoma Development

Cell signalling pathways govern physiological functions of the brain by intracellular reactions, neurotransmitter release, cell-surface receptor activation, chemical modifications of the surface proteins, transcription factors, synaptic transmission and plasticity, which are essential for brain function. Alteration of any molecular cross-talk may induce hyperactivation or dysfunction of the key molecular programmes of the neurons and brain. Innovative strategies for GB management can be developed by elucidating the molecular pathways involved in GB progression.

Mutations in genetic makeup and irregularities in major signalling pathways are primarily responsible for the onset of any malignancy in the human body, wherein the grade of disease is determined by the level of anomalies. For example, astrocytomas are tumours of astrocytes (supportive cells) that are present in the spinal cord and the brain. Mutant and wild-type isocitrate dehydrogenase genes (*IDH*) are used to classify astrocytoma and GB. Astrocytoma possesses *IDH* mutant genes and can be further graded as level 2, 3 and 4. On the other hand, IDH wild-type gliomas are defined as grade 4 glioblastomas. For tumours, grading is performed based upon natural history, but for some tumours, the grading criteria and delineation of natural history are still not known [33,34].

**Figure 1:**
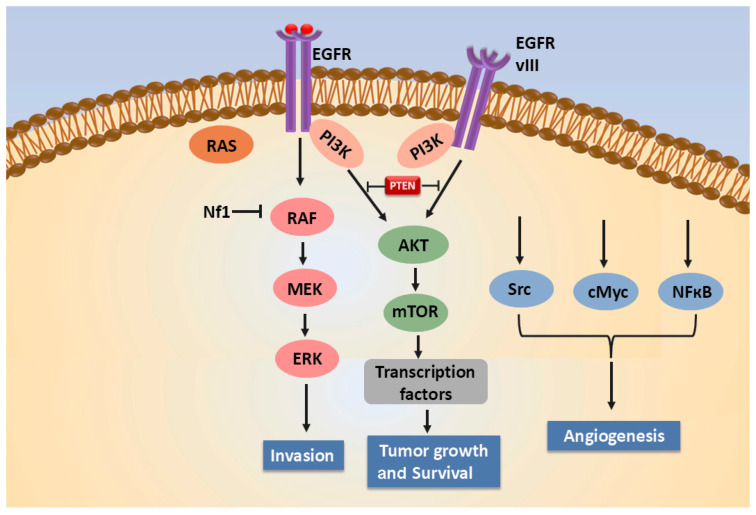
EGFR-driven oncogenic signalling pathways regulating glioblastoma growth, survival, invasion, and angiogenesis. Schematic representation of EGFR and mutant EGFRvIII signalling in GB activation of EGFR, which triggers downstream RAS–RAF–MEK–ERK signalling, promoting tumour cell invasion, while parallel activation of the PI3K–AKT–mTOR axis drives transcriptional programmes that support tumour growth and survival. Loss or inactivation of the tumour suppressors NF1 and PTEN further amplifies these pathways by relieving inhibitory constraints on RAS and PI3K signalling, respectively. EGFRvIII signalling additionally engages Src family kinases and oncogenic transcriptional regulators, including c-Myc and NF-κB, thereby enhancing pro-angiogenic signalling and tumour vascularization. Collectively, these interconnected pathways illustrate how aberrant EGFR signalling coordinates invasive behaviour, metabolic adaptation, survival, and angiogenesis in GB. Blocked lines indicate inhibitory interactions, while solid arrows denote pathway activation or signal propagation.

GB affects the cerebral hemisphere in adults and the brainstem in children. Based on its origin, GB is classified as primary and secondary. Primary GB is developed spontaneously without any prior evidence of a lower-grade tumour [35]. Secondary GB develops from pre-existing astrocytomas, where mutations in the *IDH* are commonly seen [35]. An intricate combination of multiple molecular events are involved in GB development, which include genetic mutations (EGFR, PTEN and TP53), dysregulation of signalling pathways (Wnt, TGF-β, VEGF, EGFR, CDKN2A, NF-κB, and the PI3K/AKT/mTOR), and epigenetic changes (MGMT). These molecular events critically regulate cell growth, division, survival, and migration [36]. Figure 1 highlights the role of EGFR signalling, which promotes the invasion, angiogenesis, growth and survival of GB [37]. In GB, a loss of heterozygosity has been marked in 60 to 90% of cases on the long arm of chromosome 10 [38]. In secondary GB, a mutation in the TP53 (tumour-suppressing gene) promotes cell migration, cancer cell stemness and invasion, resulting in unregulated cell growth. Furthermore, GB progression is also marked by alterations in the MDM2 and PTEN genes [39,40,41]. In primary GB, mutations in the EGFR and PDGFR genes are observed [42]. EGFR and PDGFR are tyrosine kinase (TK) receptors, where EGFR regulates proliferation, differentiation, and survival, the PDGFR gene promotes cell growth, migration, and angiogenesis. TK mutation results in the constitutive activation of receptors and the associated signalling pathways that promote tumour progression [43,44,45].

**Figure 2:**
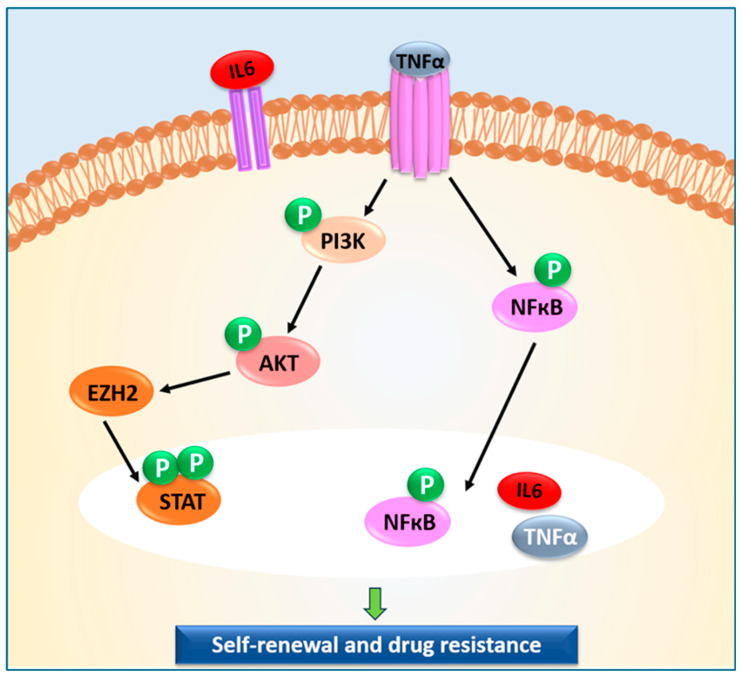
Cytokine-driven NF-κB and STAT signalling networks promoting glioblastoma self-renewal and therapeutic resistance. Schematic representation of inflammatory cytokine signalling in GB, highlighting the coordinated roles of TNFα and IL-6 in sustaining oncogenic transcriptional programmes. TNFα receptor engagement activates NF-κB signalling, leading to phosphorylation and nuclear translocation of NF-κB, where it induces expression of pro-inflammatory and pro-survival genes, including IL-6, thereby establishing a feed-forward inflammatory loop. In parallel, IL-6 signalling activates the PI3K–AKT pathway, which cooperates with the epigenetic regulator EZH2 to promote phosphorylation and activation of STAT transcription factors. Nuclear convergence of NF-κB and STAT signalling supports transcriptional programmes associated with glioma stem-like cell maintenance, self-renewal capacity, and resistance to chemotherapy and radiotherapy. Phosphorylation events are indicated by “P” symbols, and arrows denote signalling direction.

In GB development, an anomalous activation and prolonged hyperactivation of NF-κB signalling facilitates oncogenesis by tumour growth and invasion, and obstructs apoptosis (Figure 2). The pro-tumour cytokine TNFα, released by macrophages and T-cells, works at the cellular level for the activation of NF-κB signalling. Unusual activation of NF-κB keeps the target gene transcription switched on, thus modulating cellular activities like cell survival, proliferation, inflammation, and immune response [46,47,48].

Adding to genetic mutation and signalling pathways, epigenetic changes are also crucial in GB development as they regulate gene activity without affecting DNA sequence. MGMT (O6-methylguanine-DNA methyltransferase) is an important DNA repair enzyme that protects the genome against DNA-damaging alkylating agents; it removes the alkyl group from the O-6 position in guanine. MGMT serves as a biomarker for GB; H3K27 mutation in MGMT results in poor diagnosis of the tumour due to methylation of the MGMT promoter. MGMT expression and unmethylation of its promoter increase the repair power of tumours and their resistance to chemotherapeutic drugs. For the efficacy of chemotherapy drugs, methylation of the MGMT promoter and low expression of MGMT are necessary, as tumours would not undergo DNA repair [49,50]. For example, designing MGMT-targeting siRNA combined with nanoparticles could serve as an effective alternative to manage MGMT-induced resistance [51]. The synthesis of LDL (low-density lipo-protein) receptor-targeted carriers could serve as a suitable approach to block Wnt/β-catenin signalling pathways involved in the DNA repair mechanism [52]. Encapsulation of small molecules that reduce MGMT levels via epigenetic mechanisms might offer superior outcomes by minimizing MGMT-associated resistance [53].

In GB progression, nearby neurons and astrocytes are actively involved, either to support or oppose tumour development and maturation. In the first stage, GB spreads in the brain with the help of existing blood vessels. Astrocytes remain in close vicinity of the GB to provide structural and nutritional support via the blood vessels. No angiogenesis occurs during this stage. Though GB does not directly affect neurons, its close association with blood vessels exposes neurons to a nutrient-compromised and signalling-disrupted state as the metabolites and oxygen flux divert to support tumour growth. In the next stage, as the tumour advances, it invades the blood vessels, where astrocytes play an important role in balancing the invasion of GB via the release of pro-invasive factors as well as by preserving vascular integrity. Due to blood vessel invasion, localized hypoxia, and excitotoxic damage, functional impairment in neurons occurs. In the advanced/last stage, GB progression breaks the BBB, causes infiltration of immune cells and harmful substances in the brain and facilitates oxygen and nutrient supply to the tumour. At this point, astrocytes lose their protective role and neurons experience oxidative stress and impaired synaptic function, which contribute to neuronal deficit [54]. A comprehensive understanding of the molecular and cellular pathways/events, including the epigenetic changes in glioblastoma, is crucial in deciding the treatment strategy.

## 3. Blood–Brain Barrier (BBB) Alteration and Targeting Delivery Strategies

The BBB regulates the transport of essential nutrients like glucose, amino acids, iron ions, and vitamins from the blood to the central nervous system (CNS) and extracellular fluids [31]. The BBB is composed of the capillary endothelium, pericytes, basal lamina, immune cells, and astrocyte end feet. Its selective capillary network, with a diameter of approximately 7.5 μm, transports blood to each brain cell and protects the brain’s microenvironment from neurotoxins [55]. The highly selective ultrastructure of the BBB restricts the permeation of macro and micro therapeutic agents, restraining their efficacy against life-threatening diseases like GB [55].

**Figure 3:**
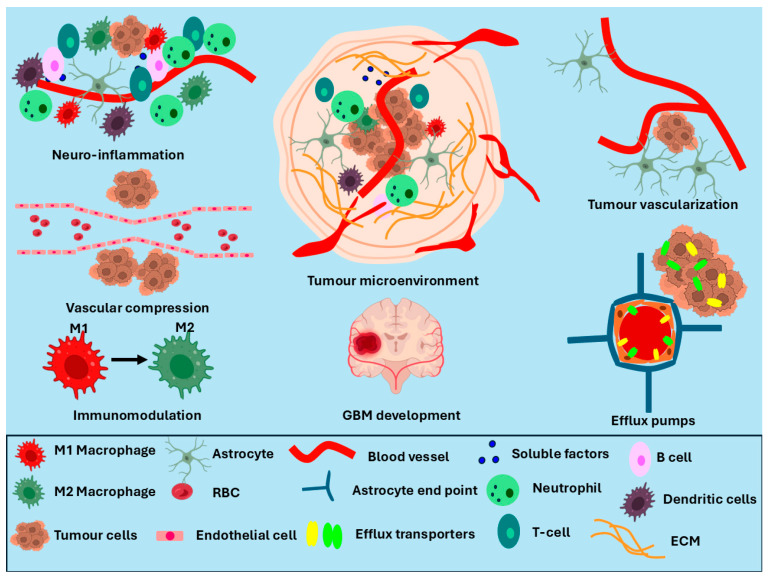
Tumour microenvironment-driven barriers that limit therapeutic efficacy in glioblastoma. Schematic representation of the major cellular, vascular, and immunological challenges imposed by the GB tumour microenvironment. GB progression is associated with profound neuro-inflammation, characterized by infiltration of immune cells and secretion of soluble inflammatory mediators that promote tumour growth and immune dysfunction. Abnormal and dysfunctional tumour vasculature leads to vascular compression, impaired perfusion, and heterogeneous oxygen and nutrient distribution, further exacerbating hypoxia and therapeutic resistance. The tumour microenvironment actively drives immunomodulation, including polarization of macrophages from a pro-inflammatory M1 phenotype toward an immunosuppressive M2 phenotype, thereby facilitating immune evasion. Concurrently, tumour-induced vascularization supports sustained growth while remaining structurally and functionally abnormal. GB cells also upregulate efflux transporters, limiting intracellular drug accumulation and reducing the efficacy of chemotherapeutic agents. Interactions among tumour cells, astrocytes, immune cells, endothelial cells, and extracellular matrix components collectively shape a dynamic and heterogeneous microenvironment that promotes GB development, progression, and resistance to therapy. Symbols denote distinct cellular populations, vascular structures, extracellular matrix elements, and soluble factors contributing to microenvironmental complexity.

Tumour progression in GB leads to the formation of another physiological barrier known as the blood–tumour barrier (BTB), which disturbs the homeostasis of the brain (Figure 3) [56]. Altered endothelial cells in the BTB promote non-uniform permeability around the tumour cells [56]. Tumour progression compresses the blood vessels, resulting in leakage in the tumour area and stimulating a heterogeneous microenvironment around the BTB (Figure 3) [56]. Activation of the hypoxia-inducible factor-1α (HIF-1α) signalling pathway in the tumour core upregulates vascular endothelial growth factor (VEGF) to promote angiogenesis [57]. The formation of abnormal blood vessels due to angiogenesis enhances permeability in the BBB and promotes a neuro-inflammatory response, leading to complications in drug delivery. Furthermore, the presence of M2 macrophages in the GB microenvironment upgrades tumour progression and hinders anti-tumour-agent delivery [56]. Importantly, it was also reported that GB progression maintains the integrity of the BBB and restricts the penetration of anti-tumour agents.

As the BBB and BTB have different biological niches, the physicochemical properties of drug delivery systems targeting these barriers might also differ. For example, bradykinin activates B2 receptors situated on the abluminal side of the BBB. This activation leads to BBB permeability and can be used to target the BBB [58]. Engineering analogues of bradykinin exhibiting high affinity for B2 receptors could enhance the BBB permeability to deliver the payload. However, hyper BBB disruption via B2 receptor activation may cause vasogenic edema and neuro-inflammation [59]. Similarly, targeting adenosine receptors expressed in luminal and abluminal surfaces of the BBB may enhance BBB permeability. Small phospholipid signalling molecules interact with GPCRs to govern vital signalling pathways. Sphingosine-1-Phosphate Receptor 1 (S1P1) was widely studied for its function in modulating BBB permeability. Previous studies highlighted that the antagonist of S1P1 could promote selective and reversible permeability of the BBB. This strategy could pave the way for delivering small molecules into the BBB [60]. Efforts have been made to improve the permeability of the BBB to deliver higher drug concentrations into the tumour environment. Anti-tumour agents coupled with vasoactive compounds or hypertonic solutions can enhance BBB permeability and improve the efficiency of drug delivery systems [61,62,63]. Efflux transporters expressed in the BBB play a prime role in eliminating drug molecules. This leads to the development of multidrug resistance [64]. Chemotherapeutic drugs such as Dox and paclitaxel induce overexpression of efflux transporters, resulting in multidrug resistance (MDR). Inhibition of these transporters could serve as an effective strategy for BBB penetration.

The development of primary and metastatic tumours disrupts the BBB and forms the BTB. It enhances permeability in tumour-associated capillaries. However, diffusion of polar molecules and large molecules across the BTB still remains a bottleneck in pharmaceutical science. Growth factor receptors, playing an important role in cancer cell infiltration, could serve as a crucial target to enhance BTB diffusion. For example, EGFR-specific peptides and aptamers conjugated with chemotherapeutic molecules may enhance transcytosis to target the BTB [65]. Scavenger receptors (SRs) are transmembrane soluble proteins capable of binding a broad range of ligand molecules, including spherical nucleic acids (SNAs). SRs are expressed in endothelial cells, macrophages, and DCs. Synthesizing nanoparticles coupled with SNAs could target SRs expressed in GB cells to enter the BTB/BBB [66]. A hypoxic tumour environment produces chemotactic factors, resulting in the recruitment of macrophages. Therefore, immunosuppressive strategies involving macrophages as a cellular carrier could enhance BTB permeation [67]. Cell-based drug carriers such as macrophages, exosomes and stem cells may offer promising outcomes due to their ability to penetrate the BTB/BBB. Under physiological conditions, the BBB may limit drug permeability. Coupling nanotechnology with focused ultrasound or convention-enhanced delivery may elevate drug concentration in the tumour region. Efforts should be made to limit the chances of harming healthy tissues when using focused ultrasound.

**Figure 4:**
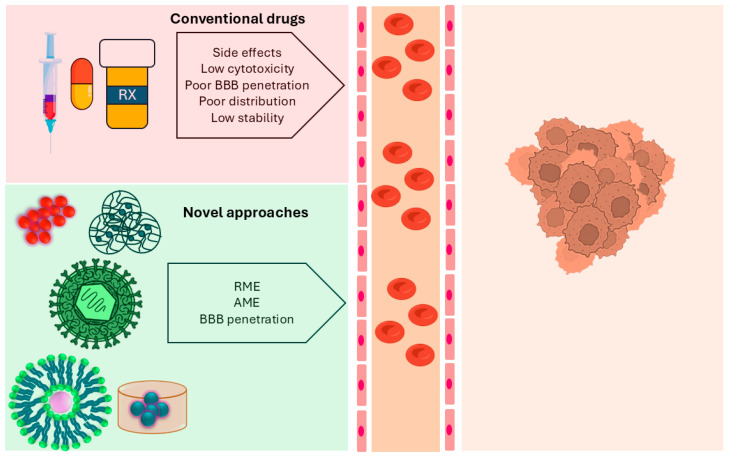
Comparative advantages of novel drug delivery systems over conventional therapeutic strategies in glioblastoma. Schematic comparison illustrating the limitations of conventional chemotherapeutic agents versus the functional advantages of advanced drug delivery platforms in GB treatment. Conventional drugs often exhibit poor BBB penetration, limited intra-tumoral distribution, low stability, and dose-limiting systemic toxicity due to non-specific absorption, metabolism, and excretion (AME). In contrast, novel drug delivery systems—including nanoparticles, liposomes, polymeric carriers, and implantable platforms—are engineered to enhance BBB penetration and tumour targeting through mechanisms such as receptor-mediated endocytosis (RME) and controlled release. These systems improve drug stability, prolong circulation time, facilitate selective accumulation at the tumour site, and reduce off-target effects. By enabling efficient transvascular transport, intracellular uptake, and sustained local drug availability, advanced delivery strategies overcome the key pharmacokinetic and biological barriers associated with conventional therapies. Collectively, this comparison highlights how rationally designed delivery systems improve therapeutic precision and efficacy in GB by integrating transport optimization with tumour-specific targeting mechanisms.

In recent years, nanocarriers functionalized with targeting peptides, ligands and antibodies have been investigated as they can bind to the cell membrane protein [68,69]. This binding of the nanocarrier with cells leads to endocytosis to release the drug molecules into the brain. Ideally, this strategy works effectively when there is high expression of the target protein on the GB cells or microvascular beds. In this regard, nanoparticles, hydrogels, catheter implants, microneedles, wafer implants, and extracellular vesicles present superior specificity and drug delivery (Figure 4).

## 4. Drug Delivery Systems for Glioblastoma Treatment

### 4.1. Nanoparticles

Nanoparticles developed from chemical synthesis display advantageous pharmaceutical properties like biocompatibility, sustained release, good encapsulation efficiency and surface characteristics. Liposomes, polymeric and metallic nanoparticles with a diameter range of 10–200 nm are primarily used in biological applications. The size, shape, physical properties and drug release behaviour of these formulations can be tailored depending on their the target tissues and environment [70,71].

Polymeric nanoparticles are composed of biocompatible polymers such as PLGA, PLA, PCL, PAMAM, etc., which minimize the toxicity associated with conventional chemotherapeutic drugs. Stable pharmacokinetics, higher bioavailability, and a larger surface area make polymeric nanoparticles a suitable candidate for GB treatment. The dimension and ligand functionalization of nanoparticles enables crossing of the BBB to reach the tumour site. Polyamidoamine dendrimer and (hyaluronic acid) HA surface-modified PLGA nanoparticles loaded with the chemotherapy drug TMZ showed enhanced BBB penetration [72]. Surface modification enables a higher drug release at an acidic pH, making it suitable for targeting the tumour microenvironment. An in vitro study demonstrated higher cellular uptake and superior cytotoxicity of the nano-formulation compared to free TMZ in the U-87 MG GB cells [72]. Incorporation of peptide-based functionalities into the polymeric formulations was found to be effective for selective binding to the GB-based cell-surface receptor. In this regard, researchers have utilized RAFT (reversible addition fragmentation chain transfer) polymerization and C1C2 peptide conjugation to target U87MG cells. Research findings suggest that peptide conjugation induces the GB-targeting feature in the formulations [73].

Another study reported that the cytotoxicity of Dox and paclitaxel against C_6_ GB cells was enhanced after their encapsulation in PLGA nanoparticles. An in vivo study performed in Wistar rats showcased higher median survival rates and enhanced overall movements [74]. Intranasal delivery of chemotherapeutic drugs enables superior solubility and internalization into the GB cells. Bioreducible polymeric micelles with PEGylated paclitaxel demonstrated superior solubility in an acidic environment to target tumour cells. The findings obtained from the study indicated that intranasal administration of polymeric micelles reduces the chances of hepatotoxicity associated with systemic paclitaxel treatment [75].

Small interfering RNA (siRNA) therapy is an emerging, suitable alternative for GB treatment; however, the stability of the nucleotides in the tumour environment hinders its clinical applications. Ionizable lipids conjugated with siRNA could offer a promising alternative to alleviate the repercussions of GB. Novel ionizable lipid (BAMPA-O16B) with siRNA downregulated target gene expression in GB and induced T-cell-dependent anti-tumour immunity [76]. Trihydrochloride ligands activate the immune system and modulate tumour genesis in GB. However, a low serum half-life, poor tumour targeting and non-specific cytokine activation are major challenges limiting their pharmaceutical applications. Cationic PEGylated liposomes encapsulating a diABZ trihydrochloride compound, a stimulator of interferon genes, enhanced the granzyme B expression (a serine protease) and increased lymphocyte infiltration in GB tissue. Results obtained from this study indicated that both intranasal and systemic liposome formulations prolonged the survival of the tumour-bearing animals [77]. Liposomal nanoparticles encapsulating the phytochemicals have also shown promising results in GB treatment. For example, liposomes encapsulating a structurally modified sphingosine induce ferroptosis in GB cells through the activation of the AMPK-mTOR-GPX4 pathway [78].

Metallic nanoparticles also offer a prominent advantage compared to conventional chemotherapeutic drugs. Nanoparticles composed of metals like gold, silver, magnesium, copper and zinc exhibit improved absorption and bioavailability. Metal nanoparticles can penetrate to the tumour sites by an enhanced permeability and retention (EPR) effect in passive targeting to kill the tumour cells [79]. A thermo-sensitive drug delivery platform coupled with chemotherapeutic drugs could facilitate higher drug release in the tumour environment. Researchers have developed thermo-sensitive liposomes coupled with gold nanoparticles and TMZ hexadecyl ester, a TMZ derivative, to target GB cells. Infrared irradiation at 780 nm elevated the temperature of the formulation to 42.2 °C and facilitated higher release of the therapeutic molecule [80].

Ultrasmall gold nanoparticles loaded with Dox and AlexaFluor-647-cadaverine penetrated the BBB, whereas pure Dox and AlexaFluor-647-cadaverine could not pass the BBB [81]. Silver nanoparticles synthesized using *Podocarpus macrophyllus* leaf extract as reducing agent showed effective anti-oxidant and anti-inflammatory activity. Furthermore, computational analysis suggested these nanoparticles may interact with NOTCH2 genes, which are upregulated in GB [82]. Another study coupled Dox-loaded Fe^2+^ nanoparticles with liposomes to enhance apoptosis and ferroptosis in GB treatment. The higher photothermal responsiveness of this formulation enhanced ROS generation and translocation of Dox across the BBB [83]. The nano-dimensional architecture of the metallic nanoparticles enables higher BBB penetration and delivers the therapeutic agent to the tumour microenvironment.

Cancer stem cells, metabolic reprogramming, tumour heterogeneity and DNA repair mechanisms in GB cells reduce radiotherapy sensitivity. This challenge can be addressed by coupling nanoparticles with radiotherapy. In this direction, copper oxide nanoparticles coated with bovine serum albumin, combined with X-ray irradiation, significantly enhanced the radio-sensitivity of U87-MG cells, leading to decreased clonogenic survival and increased apoptotic cell death compared to irradiation alone [84]. Chiarelli et al. designed iron oxide nanoparticles that promote energy transfer in photon radiotherapy. Results obtained from the in vivo study showed an intravenous dose of iron oxide nanoparticles before radiotherapy produced a three-fold reduction in tumour growth [85]. Cerium oxide nanoparticles stabilized with leaf extract demonstrated promising in vitro TMZ delivery to U-87 MG cells. Cerium oxide nanocarriers facilitated higher antiproliferative activity of TMZ compared to a pure TMZ drug [86]. Sodium-doped zinc oxide nanoparticles promoted apoptosis in U-87 MG cells by upregulating expressions of the TP53, PTEN, BAX and P21 genes [87]. Iron oxide–chitosan magnetic nanoparticles loaded with atorvastatin showed excellent anti-tumour activity in the presence of C6 cells. This formulation downregulated the expression of the Bcl-2, VEGF, and BDNF genes, which promote tumour growth [88].

Researchers have comprehensively explored the therapeutic efficacy of different nanoparticle-based formulations, which have been compiled and presented in Table 1. Though metallic nanoparticles offer significant advantages in GB treatment, their adverse effects cannot be ignored. The synthesis of metal nanoparticles often requires cytotoxic compounds; therefore, protocols should be designed to remove the unreacted or excess chemicals used during the process. Due to the nano size of the metallic nanoparticles, they have a higher circulation time and may accumulate in the liver and spleen, resulting in hepatotoxicity and organ failure [89]. Nanoparticles could promote an M2-like phenotypic shift in macrophages. Nanoparticles with high surface charge and size might be recognized as a foreign body and phagocytosed by M2 macrophages. This may lead to the release of IL-10 and TGF-β, resulting in tumour progression and resistance. Therefore, reprogramming of M2 immunosuppressive macrophages into pro-inflammatory M1 macrophages could be an effective strategy to target GB [90]. These challenges should be considered before designing effective metallic nanoparticles for GB treatment.

### 4.2. Hydrogels

The three-dimensional architecture of hydrogels supported with a cross-linked hydrophilic polymeric matrix enables higher water-retention capability. Hydrophilic functional groups such as –OH, –SO_3_H, –COOH, –CONH–, –NH_2_, etc., enhance the swelling property and structural integrity of the hydrogels in an aqueous environment [105]. By mimicking the three-dimensional structure of the extracellular matrix (ECM) and tumour microenvironment, hydrogels derived from biocompatible polymers offer an effective approach for localized drug delivery to treat GB [106]. The physical properties of hydrogels promote in situ drug delivery by passing the BBB and facilitate local drug accumulation at the tumour site [107]. Hydrogels composed of chitosan, collagen, HA, PVA, PEG, PLGA, etc., have been comprehensively studied for their suitability for GB treatment. Hydrogels can be classified as natural, synthetic and hybrid based on their polymeric constitution. For example, hydrogels made up of synthetic polymers such as PVA, PLLA, PLGA and PLA belong to synthetic hydrogels, whereas hydrogels composed of natural polysaccharides and proteins like chitosan, gelatin and collagen, etc., can be regarded as natural hydrogels. The mechanical and biological properties of the hydrogels are often enhanced when both natural and synthetic polymers are used in their synthesis process; these hydrogels are regarded as hybrid hydrogels.

PVA polymers are a suitable candidate in drug delivery due to their hydrophilic and biocompatible nature. PVA hydrogels loaded with phyto-molecule luteolin nanoparticles downregulated the epithelial–mesenchymal transition (EMT) signalling pathway, which is crucial for GB progression [108]. Phytochemicals often exhibit limited biomedical applicability due to rapid clearance and inadequate systemic circulation. Encapsulation within polymeric hydrogels offers a strategy to stabilize these compounds and enhance their bioavailability. Modifications in the functional groups of the polymers could introduce thermo-responsive behaviour in hydrogels to promote the sustained release of the drug molecules. Researchers modified the end group of Pluronic F-127 from hydroxyl to aldehyde, which enhanced the thermo-responsive property of the F-127/chitosan hydrogels. These modifications facilitated the sustained release of Dox and BMS-1 [109]. Hydrogels mimicking the secondary functions of lymphoid organs like lymph nodes could pave the way to build a new therapeutic strategy for GB treatment. Aldehyde-functionalized polyethylene glycol (PEG-CHO) and PAMAM polymers were utilized to synthesize lymph-node-mimicking hydrogels encapsulated with immune-adjuvant Dox and siCD73. Due to the proton sponge effect, the hydrogels escaped the lysozyme and released siCD73, thereby enhancing the immune response. Immune-adjuvant functionalization activated DCs and promoted antigen presentation [110].

The advancement of innovative drug delivery systems that encapsulate chemotherapeutic agents, siRNA, and immune adjuvants has the potential to address GB through a multi-faceted approach. Apart from drug delivery vehicles, hydrogels may be used to trap remaining GB cells after tumour surgery. A tumour recurrence chance cannot be avoided as the remaining GB cells could invade the adjacent healthy tissues, resulting in challenges in residual radiotherapy and chemotherapy. A hydrogel composed of sodium alginate, chitosan and genipin as a cross-linker possessed a macro-porous ultrastructure and displayed the compressive strength of brain tissues. This investigation illustrated that hydrogels cross-linked with genipin possess the capability to sequester F98 glioma cells while maintaining their physicochemical characteristics following exposure to radiation dosages comparable to contemporary therapeutic approaches for GB [111].

Hydrogels that exhibit sensitivity to both physical and chemical stimuli may serve as an effective candidate for localized drug delivery, thereby reducing tumour proliferation following surgical intervention. Elevated levels of reactive oxygen species (ROS) present at the residual tumour site, post-surgery, can be exploited as a strategic target for the administration of therapeutic agents. Investigators have encapsulated bis(2-chloroethyl) nitrosourea and TMZ within ROS-sensitive PLGA nanoparticles and subsequently integrated this entire construct into thermo-responsive hydrogels formulated from chitosan, gelatin, and β-glycerophosphate. After the resection of over 90% of the glioblastoma tissue, the dual-sensitive hydrogel drug delivery system was administered into the surgical cavity. The median survival duration of the experimental groups treated with the hydrogel system significantly increased compared to the untreated negative control group [112].

Another study targeted both GB stem cells and GB cells by encapsulating GemC_12_ lipid nanocapsule hydrogels, salinomycin and curcumin. Nanocapsule-based hydrogels effectively released the drug molecules for up to 30 days and maintained their stability for six months [113]. Co-encapsulation of pharmaceuticals targeting stem cells within the GemC_12_-LNC hydrogel has the potential to diminish both GB cells and glioblastoma stem cells, thereby suggesting a promising avenue to prevent the emergence of GB relapses. Table 2 highlights some of the hydrogel formulations used to treat GB.

Hypoxia, an acidic pH and altered metabolic activity in the tumour microenvironment could suppress immunity and promote tumour growth [114]. Therefore, designing a therapeutic strategy that can alter the immunosuppressive tumour microenvironment into an active immune environment can mitigate tumour progression. *Clostridia* bacteria species grow only in hypoxic conditions and produce toxins that are cytotoxic to the tumour cells. Also, it was observed that *Clostridia* could facilitate the immune response and enhance the anti-tumour activity of chemotherapeutic drugs. Researchers exploited this feature of the *Clostridia* strain, C-novyi-NT, to change the immunosuppressive GB tumour microenvironment into an active immune environment [115]. Spores of C-novyi-NT and metformin loaded into melittin-RADA_32_ peptide hydrogel activated CD8^+^ T-cell responses, macrophage polarization and infiltration of DCs and NK cells [115]. This study elucidated that a combination of biological agents like *Clostridium* species with chemotherapeutic drugs could promote the apoptosis of tumour cells through a multi-faceted pathway.

Hydrogels exhibit outstanding biocompatibility, low cost, flexibility and exceptional drug loading capabilities, allowing for precise administration at localized sites. Consequently, in comparison to traditional drug delivery systems, the clinical response rates and anti-cancer efficacy achieved through hydrogel delivery systems are frequently superior. Nonetheless, several challenges have persisted regarding the clinical translation of hydrogels in recent years. The quality control associated with large-scale production constitutes a major impediment during the translational process. Different quality attributes such as dose uniformity, stability, pH, visual appearance, in vitro drug release and apparent viscosity should be meticulously regulated throughout the mass synthesis of hydrogel delivery systems.

**Table 2:** Different strategies used in GB treatment.

Formulation/Strategy	Drug or Therapeutic Agent	Observations	Reference
Pluronic F127 and HA	Dox	Radiation-triggered drug release Drug retention enhanced	[116]
Dextran and collagen hydrogels with meso-porous polydopamine nanoparticles	TMZ	Enhanced intra-tumoural retention Apoptosis induction Overcomes TMZ resistance	[117]
Oxidized high-amylose starch	Macrophage and BLZ945	Induces macrophage polarization and apoptosis Slow release of drug	[118]
Silk fibroin hydrogels and lipid NP combination	Dox and ibuprofen	Cytotoxic activity against C6 glioma cells	[119]
Exosomes	miR-7-5p and exosomes	Reduced GB cell proliferation	[120]
Exosomes	Plasmid DNA	Expression of Herpes simplex virus thymidine kinase (HSVtk) gene Apoptosis induction	[121]
Exosomes	Adjuvant CpG	Activation of DCs and T-cell response generation	[122]
Exosomes	Dox	Accumulation of drug in GB	[123]
Oncolytic virus	Oncolytic herpes simplex virus	Activated natural killer cells and T-cells	[124]
Micro-bubbles via low-intensity focused ultrasound	Paclitaxel	High encapsulation efficiency Suppressed tumour growth	[125]
Self-assembled micelle	siRNA	Disruption of the cellular energy metabolism of GB cells Mitochondrial dysfunction	[126]
Peptide–oligonucleotide composite nanotubes	Dox	70% reduction in tumour mass Enhanced DNA damage, oxidative stress and reduced proliferation	[127]

### 4.3. Extracellular Vesicles

Extracellular vesicles are natural nano-sized lipid particles secreted by numerous cell types such as cancer cell lines, mesenchymal stem cells (MSCs) and neural stem cells (NSCs). They can be classified as exosomes, ectosomes, and apoptotic bodies. Exosomes, with a diameter ranging between 30 and 150 nm, have been comprehensively studied in GB treatment. The superior drug loading efficacy, BBB transfer capability, and biocompatibility of exosomes enable the restoration of the anti-cancer drug chemosensitivity in glioma tissues [128,129].

Preclinical investigations project rapamycin as an excellent drug candidate in GB treatment; however, its poor BBB penetration limits therapeutic potency in the tumour microenvironment. Encapsulation of rapamycin in exosomes with a size of 30–160 nm promoted higher drug release in the tumour environment and decreased the angiogenesis of GB through the VEGF-VEGFR axis. It was reported that due to exosome encapsulation, rapamycin penetrated the BBB, resulting in cell cycle arrest at the G1 phase in GL261 GB cells [130].

Antibody drugs are utilized to treat a broad range of cancers, including breast, colon, small lung cancers and GB, owing to their long serum half-life and high specificity. Bevacizumab (BEV) is the only antibody drug used to treat GB. It kills tumour cells by limiting angiogenesis and inhibiting VEGF. Several clinical trials showed poor BBB penetration of BEV, lowering its cytotoxicity towards GB. To address this challenge, BEV is loaded into exosomes derived from glioma C6 cells. Results showed that exosomes delivered the BEV drug into the tumour site by penetrating the BBB and induced apoptosis in the glioma cells [131]. Exosomes derived from glioma cells have similar biological features to their native cancer cells. This property may enhance the tumour-targeting efficiency of glioma-derived exosomes compared to non-glioma-derived exosomes. A study performed by Lee et al. isolated exosomes from glioblastoma cells (U87MG) and loaded the anti-cancer selumetinib drug into them. This formulation was found to be non-toxic to the healthy brain cells, whereas it inhibited the proliferation of U87MG cells [132].

Advancements in immunotherapy and molecular biology have developed peptide-, viral vector-, dendritic cell- and mRNA-based vaccine platforms for GB treatment. However, the complex synthesis process, poor antigen loading and low immune response activation are major challenges associated with these platforms [133,134,135]. These challenges could be addressed by isolating exosomes from the GB cells, which do not require antigen loading to activate the immune system. A recent study showed that homologous exosomes derived from GB tumour cells enhanced TNF-α, IL-6, and IFN-γ expression, resulting in innate immunity activation. Furthermore, this vaccine platform increased mature dendritic cells, cytotoxic T-cells, and the memory T-cell population [133].

EGFRvIII overexpression in the GB cells is linked with aggressive tumour progression, amplified invasiveness and chemotherapy resistance. Therefore, targeting EGFRvIII could enhance the therapeutic efficacy of the biological agents against the GB cells. Researchers isolated exosomes from mesenchymal stem cells (MSCs) presenting the antibody EGFRvIII, which were co-loaded with apoptosis-inducing gene therapy agents, cytosine deaminase (CDA) and miR-34a. Incorporation of the antibody EGFRvIII into the exosomes augmented their specificity towards EGFRvIII-positive GB cells, resulting in apoptosis in the cancer cells [136]. Apart from antibody conjugation, aptamers also facilitate specific uptake of exosomes by GB cells. Researchers conjugated Angiopep-2 and CD133 RNA aptamers to exosomes via an amphiphilic molecule bridge. Due to aptamer functionalization, nano-formulations are efficiently accumulated in U87MG and GSCs. This treatment inhibited the proliferation of GSCs and U87MG cells and extended the median survival time of tumour-bearing mice [137]. The roles of a few of the exosome formulations are also summarized in Table 2.

Exosome research has progressed remarkably in GB treatment, but there are certain challenges that need to be examined to develop an efficient treatment strategy. Efforts need to be focused on optimizing the isolation of pure exosomes with minimal or no contamination of microvesicles and apoptotic bodies. Clinical trials require the large-scale production of exosomes, which is also a significant pitfall in GB therapy. The most difficult question in exosome research is that of their alteration in efficacy due to their heterogeneity in size, shape, and origin [138].

### 4.4. Microneedles

The route of administration plays a significant role in determining the drug concentration around the tumour site. Orally administered anti-cancer drugs go through the hepatic first-pass metabolism and enzymatic degradation, causing limited BBB penetration and poor bioavailability. Similarly, the parenteral route of administration requires high cost and trained medical personnel with lower brain-targeting efficiency. On the other hand, the intranasal route faces challenges due to low drug volume, nasal irritation and mucociliary clearance [139]. Dissolving microneedles composed of biodegradable polymers such as chitosan, maltose, silk, cellulose, PVP, PLGA, PVA, etc., offer high drug loading, sustained release and low systemic side effects [140]. Dissolving microneedles can be applied by the transdermal and intranasal routes to deliver drug molecules into the brain. The intranasal route of administration of dissolving microneedles holds promise due to high mucosal barrier permeation and low ciliary clearance [139,141,142]. It results in high BBB permeation and tumour targeting and minimizes the chance of side effects [142].

Researchers have developed microneedle formulations using silk, gelatin, hyaluronic acid, and PVP, loaded with drugs such as cinnarizine, TMZ, and bevacizumab [143,144,145]. These microneedle-based systems showed promising results in reducing tumour volume with improved therapeutic effects. For example, a silk fibroin-based microneedle patch loaded with TMZ and bevacizumab enhanced apoptosis and limited angiogenesis. A microneedle-based formulation improved the mean survival rate of diseased mice by controlling tumour growth [143]. To achieve sustained drug release in the tumour microenvironment, researchers chemically modified gelatin methacryloyl and used it for synthesizing oligonucleotide (CpG)-coupled microneedles. The formulation targeted glioblastoma-associated macrophages. Researchers found that CpG is responsible for attracting dendritic cells and glioblastoma-associated macrophages to reprogram the immunosuppressive microenvironment of GB [144]. The results obtained from this study could help in developing an alternative strategy to eradicate the residual GB cells after surgery, which augments the risks of tumour recurrence.

HA and bovine serum albumin were used to synthesize TMZ- and niclosamide (NIC)-loaded microneedles to overcome TMZ resistance in GB. Prolonged drug release from the microneedles inhibited the activity of O6-methylguanine-DNA methyltransferase and promoted TMZ activity. An in vivo study showed excellent anti-tumour activity of the drug carriers due to extended survival time in orthotopic tumour-bearing mice [145]. Similarly, methacrylated hyaluronic acid microneedle patches loaded with TMZ and siPLCG1-siRNA showed promising outcomes in GB management. Utilization of siRNA inhibited the tumour growth by decreasing STAT3 gene expression. Additionally, TMZ damaged the DNA of cancer cells, leading to apoptosis [146].

Combinational treatment involving photodynamic therapy and protein degradation could augment apoptosis in GB. A previous study found that the inhibition of bromodomain-containing protein 4 (BRD4) in GB limits tumour progression [147,148]. Therefore, researchers employed Proteolysis-Targeting Chimera (PROTAC) nanoparticles and self-oxygenating BSA-MnO_2_ (BM) nanoparticles to degrade BRD4 proteins. Microneedles rapidly released PROTAC at the tumour site, where PROTAC was activated due to the acidic tumour microenvironment. Furthermore, near-infrared light activates singlet oxygen production, thereby triggering BRD4 degradation. BM nanoparticles at the inner layer of the microneedle acted as an oxygen-generating station to respond to the hypoxic tumour environment. BM nanoparticles converted H_2_O_2_ into O_2_ and facilitated the activity of PROTAC. A subcutaneous and orthotopic GB tumour model verified the tumour inhibition activity of nanoparticle-loaded microneedles [149].

Microneedles are designed to remain at the target site to deliver the drug molecule locally. Therefore, maintaining microchannel integrity throughout the delivery process is the major challenge associated with it. As microneedles are often implanted at the tumour site, steps should be taken in designing effective sterilization techniques for their implantation. Extensive in vitro and in vivo tests should be conducted to generate product-specific efficacy and safety data for microneedle formulations. As microneedles are constructed using biocompatible polymers, their long-term drug stability, shelf life, drug degradation, and mechanical stability may require comprehensive analysis before their use for complex biological applications like GB treatment [150].

### 4.5. Other Drug Delivery Agents

Apart from the previously discussed drug delivery systems, oncolytic viruses, wafer implants, bioresorbable electronic patches and catheter implants are some of the vital strategies used in GB treatment. Oncolytic viruses stimulate the apoptosis of tumour cells by activating immune functions. Research involving Myxoma, pox virus, BoHV-4, polio virus (PV), herpes simplex virus (HSV), Newcastle disease virus (NDV), Zika virus, M1, etc., demonstrated encouraging results in GB treatment [151,152,153,154]. Limited spread of viruses inside the tumour is one of the major challenges in oncolytic virus strategies. Therefore, designing a suitable carrier for targeting the tumour site is crucial. Researchers demonstrated chimeric pox virus CF-17 carried by neural progenitor cells exerts a growth-limiting effect on GB stem cells. In vitro and in vivo experiments confirmed the superior tumour targeting and anti-tumour activity of the CF-17 virus with neural progenitor carriers compared to the pure form [155]. Similarly, the low tumour-targeting efficiency of conventional oncolytic adenoviruses was elevated by surface modification. Cholesterol surface modification of oncolytic adenovirus induces the adsorption of apolipoprotein E from the blood and penetrates the BBB. After penetrating the BBB, the adenovirus induces an immune response against GB by recruiting DCs and CD8^+^ T-cells [156].

Herpes simplex virus C5252 armed with anti-programmed cell death protein 1 antibodies and interleukin-12 induced apoptosis in GB cells. The 5252 virus facilitates caspase-3/7 activation via downregulating ciliary neurotrophic factor receptor α, resulting in the apoptosis in GB cells [157]. Profound immunosuppression was observed in the tumour microenvironment of GB. This leads to poor activity of tumour-infiltrating lymphocytes and CD8^+^ T-cells, which protect the host from cancer. Therefore, strategies that can reverse immunosuppression in GB could provide better outcomes. Oncolytic Zika virus treatment promoted activation of the type I interferon signalling pathway in GB cells. It was observed that the Zika virus treatment exerted excellent CD4^+^ and CD8^+^ T-cell infiltration [158]. Neutralizing antibodies present in the host often degrade the oncolytic virus and reduce their efficacy against GB. Designing a virus carrier that can evade neutralizing antibodies could enhance the effect of the oncolytic virus. Therefore, researchers replaced capsid protein HVRs from the serotype 5-based Delta-24-RGD virus. The resulting chimeric virus, Delta-24-RGD-H43m, evaded neutralizing anti-Ad5 antibodies and conferred a higher rate of long-term survival of tumour-bearing mice compared to the Delta-24-RGD virus [159].

Newly diagnosed and recurrent gliomas could be treated with carmustine wafers. This product is marketed as Gliadel^®^ and is made with biodegradable polymers with the alkylating agent carmustine (Bis-ChloroethylNitrosoUrea (BCNU)). Its clinical application is still in infancy due to its high cost, sterility issues and design failure due to absorption of plasma proteins on its surface after implantation. Previous reports suggested its efficacy in MGMT-methylated glioblastomas and highlighted that more studies should be designed to analyze its activity against high-grade gliomas [160]. The cerebraca wafer containing the drug molecule (Z)-n-butylidenephthalide (BP) eliminated the residual tumour after glioma resection. The BP activated T-cell cytotoxicity and inhibited PD-L1 expression to inhibit tumour progression [161]. To improve the outcome in newly diagnosed GB patients in Japan, a prospective phase II clinical trial was conducted between October 2015 and April 2018. It showed that inplantation of carmustine wafers followed by radiation, bevacizumab and TMZ treatment may enhance the survival of Japanese glioblastoma patients with maximal resection [162].

Bioresorbable electronic patches consist of a biocompatible polymeric drug reservoir and a wireless electronic device. After its implantation at the tumour resection site, the electronic device can be stimulated wirelessly to produce a mild thermal effect to stimulate drug release from the polymeric reservoir [163]. A flexible and sticky wireless device made up of biodegradable polymers such as PMMA, PLA, PLGA, etc., showed efficient delivery of Dox, minimizing drug leakage to the cerebrospinal fluid. In vivo experiments revealed tumour suppression and the prolonged survival of animals treated with this device [164].

Convection-enhanced delivery (CED) uses catheters that are connected to pumps to provide a continuous micro-infusion of desired drug molecules to the target tissues. This strategy enables drug distribution within the central nervous system and bypasses challenges posed by the BBB. It minimizes dose-related systemic toxicities and delivers the drug molecules at higher concentrations [165]. For example, topotecan is a cytotoxic drug to glioma cells, but its clinical applications are limited due to poor brain penetration and toxicity. Researchers designed a catheter pump system to deliver topotecan for a longer duration of time. A clinical trial performed in five patients revealed that topotecan significantly inhibited tumour proliferation [166].

The treatment outcomes of the combined approach using CED and nanomaterials showed promising results in GB therapy. Nanoantenna coated with triphenylphosphine-conjugated polyglutathione generated ROS in mitochondria, resulting in dendritic cell activation and T-cell recruitment at the tumour site. This study used CED to improve the tumour penetration ability of nano-formulations [167]. In another study, researchers used a nanoparticle-mediated CED approach to address TMZ resistance. This study showed that nanoparticles encapsulating an oxaliplatin prodrug and a DNA intercalator inhibit the growth of TMZ-resistant cells derived from patients’ xenografts [168]. Xi et al. delivered nanodiamond loaded with Dox by CED. This approach enhanced the cellular uptake of the nanodiamond formulation compared to the pure Dox in C6 and U251MG cells [169]. CED of a DNA nanocarrier encapsulating RP-182 peptides targeted M2 tumour-associated macrophages and reprogrammed them into an anti-tumour M1-like phenotype. This approach might be helpful for brainstem gliomas in future [170]. Another study integrated the microtubule inhibitor monomethyl auristatin-F with lipid head groups and conjugated it with poly-l-glutamate and PEG to form a nano-formulation. CED was employed to deliver the nano-formulation, resulting in higher tumour penetration and drug activity [171].

Backflow is a major challenge associated with CED. Backflow occurs when the infusate flows along the catheter insertion tract rather than targeted tissues. It lowers the intended dose and may cause toxicity [165]. The success of CED-based delivery depends on the optimal placement of catheters. Suboptimal placement of the catheter may result in a risk of toxicity due to leakage of molecules into the blood or cerebrospinal fluid. Therefore, the development of image-guided systems that can place catheters optimally could enhance the outcomes of CED [172]. Indeed, CED could enable the targeted delivery of therapeutic payload to the targeted tissues; however, the effect of this drug delivery strategy could be comprehensively studied with larger patient populations for better insight.

Focused ultrasound (FUS) is a non-invasive energy-delivery method that can deliver acoustic waves into the targeted tissues. Rapid advancements in FUS enable precise modification of TME and facilitate BBB opening through acoustic waves. Several phase I clinical trials have already entered phase 2 due to promising results in patient survival time due to the FUS strategy. It has reduced the challenges associated with the BBB/BTB by enhancing transcytosis and intra-tumoural drug distribution [173]. FUS has evolved over time by its combination with micro-bubble-assisted systems. In the FUS–micro-bubble strategy, micro-bubbles are delivered to the bloodstream and FUS induces oscillations in the micro-bubbles, resulting in reversible opening of the BBB [173]. The frequency of the ultrasound waves, micro-bubble oscillations and size of the formulation play a major role in this strategy. For example, researchers attempted to optimize the parameters of FUS to generate high-amplitude oscillations in micro-bubbles to deliver lipid nanoparticles. In this study, different frequencies of 80, 250 and 850 kHz were utilized to deliver siRNA-Cy5-lipid nanoparticles. In vivo experiments showed that a frequency of 850 kHz and a 180 kPa pressure exerted a safe opening of the BBB to deliver the formulations [174]. A combinational approach was developed using nano- and micro-bubbles. Researchers synthesized gambogic acid-loaded PLGA nano-bubbles and conjugated them with cationic lipid bubbles. Intravenous administration of gambogic acid nano-/micro-bubble formulations, with FUS assistance, enhanced the penetration of the chemotherapeutic drug gambogic acid [175]. Similarly, another chemotherapy drug, epirubicin, has been loaded into pH-responsive DNA nanostructures. An FUS–micro-bubble strategy enhanced BBB and BTB opening to deliver the drug molecule in a mouse model bearing an intracranial glioma xenograft. FUS–micro-bubble mediation produced a 4.4-fold increase in drug delivery compared to unsonicated animals [176]. Industrially produced micro-bubbles may cause cytotoxicity, resulting in immune rejection. Therefore, micro-bubbles produced from cerebral microvessel cells could mitigate this concern. Building on this concept, iron oxide was coupled with Dox embedded in biomimetic micro-bubbles extracted from cerebral microvessel cells. This formulation exhibited excellent stability and anti-tumour activity in orthotopic glioblastoma mice [177].

There are certain challenges associated with FUS that need to be addressed for better outcomes in GB treatment. For example, standardized FUS parameters may cause inconsistent responses due to tumour heterogeneity. Furthermore, patient-specific phase correction optimization may need to be performed due to acoustic aberrations in different patients [173].

The intranasal delivery approach is another key method to bypass the BBB and reduce systemic toxicity. In this method, olfactory nerve and the trigeminal pathways can be used to deliver the drug molecules to the CNS. Recently, this approach was employed to target EGFRvIII-overexpressing GBM [178]. Researchers synthesized Dox-loaded CAR-extracellular vesicle mimetic formulations from monocytes. This study highlighted that the Dox-loaded nanofoumulation facilitates higher accumulation of the drug at the GB site. Evaluation of the biodistribution and immunogenicity of the CAR-modified formulation is important in a prolonged treatment window [178,179]. Another study encapsulated paclitaxel in PLGA nanoparticles and radio-labelled it with ^125^I to study the biodistribution of the drug molecules after intranasal administration. The in vivo study unravelled that the intranasal delivery strategy resulted in a higher uptake and anti-GB efficacy [180]. TMZ-integrated gold nanoparticles were functionalized with an antibody to target ephrin type-A receptor 3. In vivo studies outlined that intranasal administration of this formulation to orthotopic-GB-bearing rats enhanced the median survival time by 42 days and enhanced tumour cell apoptosis [181]. The therapeutic activity of lomustine is reduced due to poor bioavailability and toxicity. This challenge was addressed by encapsulating lomustine and n-propyl gallate in liposomes and delivering it by the intranasal route. This approach resulted in enhanced flux and permeability coefficients of drug molecules compared to lomustine and n-propyl gallate suspensions [182]. In order to achieve targeted brain delivery, paclitaxel was loaded in ascorbic acid-conjugated polycaprolactone (PCL) nanoparticles. Free paclitaxel and paclitaxel-loaded nanoparticles were intranasally administered into male Sprague Dawley rats. A pharmacokinetic study showed the nano-formulation delivered a higher concentration of paclitaxel into the brain compared to the free paclitaxel [183]. Intranasal delivery of plasmid DNA is challenging due to the large size of DNA. To address this problem, a cell membrane dexamethasone-conjugated polyethylenimine-based nano-vesicle formulation was developed to deliver the plasmid DNA via the intranasal pathway. The cell membrane of GB cells was used in this study to reduce the surface charge of the formulation, which may enhance the delivery efficacy of the drug delivery system. An in vivo intracranial GB animal model study showed a nano-vesicle formulation had higher plasmid DNA delivery efficacy compared to other formulations [184]. An intranasal delivery system was designed using lipid carriers to deliver atorvastatin for GB treatment. An in vivo pharmacokinetic study observed a 2.7-fold increase in atorvastatin concentration in the brain via intranasal administration over the intravenous route of administration [185]. Though intranasal drug delivery provides advantages by avoiding the BBB, mucociliary clearance, the toxicity of the excipients and the physiological parameters of the delivery vehicles should be studied comprehensively to determine the safety aspect of the carriers [186].

## 5. Status of Clinical Trials

Clinical trials rigorously assess the efficacy and toxicity of the therapeutic agent to prove its suitability for human application. For example, a phase I clinical trial was designed to evaluate the safety of oncolytic adenovirus Delta24-RGD. In this study, 20 patients with recurrent glioblastoma were recruited. The oncolytic virus was locally administered by convection-enhanced delivery (CED) in the tumour and surrounding brain tissue. This study found that IFNγ and TNFα concentrations may embody a suitable biomarker for response in future oncolytic viral trials. Local administration of the oncolytic virus was found to be safe and induced a local inflammatory reaction [187]. Researchers conducted a phase I/II study (UMIN-CTR Clinical Trial Registry UMIN000002661) on 13 patients with progressive glioblastoma by administering triple-mutated oncolytic herpes virus G47∆. Herein, headache, fever and vomiting were reported as the most common adverse events. Three patients survived >46 months, and the median overall survival was 7.3 months [188].

The safety of reovirus to treat recurrent malignant gliomas in adults was evaluated by Kicielinski et al. In this study, 15 adults were treated with a dose escalation, ranging from a 1 × 10^8^ to a 1 × 10^10^ tissue culture infectious dose 50. Results obtained from this study showed reovirus to be a safe agent to treat GB [189]. To assess the efficacy of a combinational approach involving reovirus, granulocyte–macrophage colony-stimulating factor (GM-CSF) and TMZ, a phase Ib trial was conducted involving 15 patients. Intravenous administration of an oncolytic virus with GM-CSF was tolerable in 87% of patients and the median overall survival was 13.1 months [190]. The safety of recombinant polio–rhinovirus when administered intracerebrally by convection-enhanced delivery in grade 3 and 4 GB patients aged between 4 and 21 was also studied. This study showed that it is safe to use recombinant polio–rhinovirus in GB patients, with an overall median survival time of 4.1 months [191].

Apart from using oncolytic virus drug repurposing, using antivirals showed some promising results in clinical trials. A clinical trial examined median survival time when valganciclovir was used alongside standard care therapy in GB patients. Patients receiving more than 6 months of valganciclovir achieved 24.1 months of overall survival time [192,193]. Furthermore, an extended retrospective study showed patients treated with more than 6 months of valganciclovir achieved a 90% survival rate at 2 years [192,194]. A phase I clinical trial was designed to test the safety of Nelfinavir, an antiviral drug used to treat HIV/AIDS. In this study, two doses of Nelfinavir (625 mg and 1250 mg) were orally administered prior to a TMZ dose and radiotherapy. This study showed a 625 mg dose is well tolerated among patients [195]. The clinical implications of using mebendazole, an anti-parasitic drug, for treating recurrent GB were evaluated in a phase 2 trial. In this study, mebendazole was administered with lomustine and TMZ. The endpoint result showed no significant improvement in the overall survival in recurrent GB [196].

The anti-cancer activity of several targeted drugs, such as bevacizumab, nivolumab, Olaparib, cediranib, etc., was evaluated in clinical trials in recurrent GB patients. Among these, bevacizumab is widely used to treat recurrent GB. Bevacizumab is a monoclonal antibody that targets VEGF, and it was approved by the US FDA in 2009 [197]. Overall, patient survival was tested in clinical trials to evaluate the efficacy of other targeted drugs vs bevacizumab. The efficacy of nivolumab, a programmed cell death 1 immune checkpoint inhibitor, was compared with bevacizumab in recurrent GB patients. Researchers conducted phase 3 trials in 369 patients and found that nivolumab therapy did not improve overall survival compared with bevacizumab [198]. Similarly, the efficacy of olaparib and cediranib in GB patients was compared with bevacizumab. Olaparib is a poly-ADP ribose polymerase (PARP) inhibitor, and cediranib is a pan-vascular endothelial growth factor (VEGF) receptor inhibitor. A phase 2 trial was conducted between December 2017 and November 2018 in 70 adult patients with recurrent glioblastoma. Researchers combined Olaparib and cediranib drugs to investigate the survival benefit of these drugs compared to bevacizumab monotherapy. There was no significant survival benefit perceived in patients treated with cediranib/olaparib compared to patients treated with bevacizumab monotherapy [199]. Table 3 shows records of clinical trials involving a broad range of therapeutic agents used in GB management.

Phenotypic, genotypic and transcriptional heterogeneity in GB makes it difficult to design effective drug molecules that can target all grades of tumour. Additionally, the BBB serves as a barrier for several drug molecules to reach the tumour site. More phase 0 clinical trials can be designed to evaluate the BBB penetration efficacy of the drug molecules. Considering patient stratification based on molecular tumour markers may help to achieve higher median overall survival due to superior tumour targeting.

## 6. Challenges and Future Perspectives

GB exhibits cellular and genetic heterogeneity among patients or within individual tumours. It may result from genetic mutations and chromosomal structural variations, posing fundamental challenges to design a suitable drug delivery platform [31]. A patient suffering from GB may display more than one tumour cell receptor within a tumour and a single treatment approach may not be effective in eradicating the entire cell population. GB cells often overexpress drug efflux pumps to eliminate the chemotherapeutic agents and diminish their therapeutic activity [209]. Heterogeneous GB cells often accompany stem cells, which are inherently resistant to radio and chemotherapy, leading to tumour recurrence post-treatment.

The efficacy of the drug delivery agents has been evaluated using human-derived GB cell lines. Evaluation of their efficacy in the presence of patient-derived GB cells could offer better insights. Furthermore, in vivo experiments are generally conducted using immunodeficient Balb/c nude mice, which cannot replicate the complex immune environments of the body. Inconsistencies are often observed when evaluating the drug in patients due to the rapid clearance of the drug molecules by immune cells.

A drug delivery system with precision targeting and biocompatibility is essential for improving clinical outcomes. The GB microenvironment possesses hypoxic conditions, weak acidity and enhanced protease concentration. These physical attributes may be used as stimuli for localized drug delivery. Furthermore, coupling these stimuli-responsive systems, which can respond to external triggers like temperature, ultrasound, magnetic fields and light, may offer additional advantages in controlling the dynamics of the drug release. To enhance the heterogeneous GB-targeting capabilities of drug carriers, more than one ligand specific to tumour cell receptors can be added to the surface of nano drug delivery systems by chemical modification. The same strategy can be applied to neurotropic viruses and exosomes to express multiple targeting peptides using genetic engineering.

Polarization of neutrophils into tumour-associated neutrophils is one of the challenges faced in GB treatment. Neutrophils are one of the first cells to respond to an injury or disease. In GB, neutrophils get converted into a pro-tumour phenotype to promote tumour progression, angiogenesis, and metastasis. Tumour-associated neutrophils also secrete immunosuppressive cytokines and induce ROS to suppress tumour-targeting immune responses [210]. Hypoxia, nutrient deprivation, chronic exposure to tumour antigens and altered metabolic activity in the tumour microenvironment induce T-cell exhaustion. This leads to lower production of anti-cancer cytokines and upregulated expression of inhibitory “checkpoint” proteins like PD-1, TIM-3, and LAG-3 [211]. These challenges offer major obstacles to designing a suitable immune therapy for GB treatment.

New strategies can be developed utilizing a combination of exosomes, viruses and nanoparticles to enhance the cytotoxicity of the drugs against GB cells. For example, zinc sulfide-based exosome-coated nanomaterials exhibit excellent permeability across the BBB and effectively target GB cells [212]. Similarly, researchers encapsulated siRNA in exosomes and conjugated it with magnetic nanoparticles by antigen–antibody interactions. Synergy between nanoparticles and siRNA enhanced ferroptosis, cell death and tumour-targeting features to achieve multi-faceted tumour inhibition [213].

Despite significant progress in the nanotechnology field, its translation from bench to bedside is still in its infancy. In GB treatment, toxicity, off-target effects, scalability and BBB/BTB permeability are major concerns limiting clinical outcomes [214,215]. The accumulation of nanoparticles in off-target tissues may induce toxicity and cause side effects. Nanoparticles with high surface charge and poor functionalization may induce M2 macrophage induction to promote tumour progression and oxidative stress [214]. Nanocarriers with poor biodegradability remain in the system for a longer duration, may induce an inflammatory response, and can cause toxicity. The large-scale production of nanoparticles with consistent charges, sizes, loading efficacies and chemical properties is still challenging. Batch variation in loading and encapsulation efficacy may lead to poor clinical outputs, which may cause a delay in regulatory approval [214,216]. Nanoparticles possess high chemical reactivity due to a high surface-to-charge ratio. Therefore, maintaining the same physicochemical feature in different biological milieus with different physiological conditions might be challenging. It is extremely difficult for researchers to mimic the BBB/BTB in an in vitro setup. It was often noticed that nanoparticles perform better in a laboratory setup and fail in clinical studies. The presence of the BBB/BTB is one of the major hurdles in GB therapy. Though receptor-mediated transcytosis offers better BBB penetration, the heterogeneous nature of the BTB may result in irregular drug distribution, leading to poor therapeutic efficacy [214,217]. Orthotopic in vivo models mimic the tumour environment to some extent; however, it does not stimulate the complex human cancer milieu. Growth of human cancer is a spontaneous process that often takes several months to years [218]. Tumours engineered in orthotopic models often grow within weeks to meet preclinical and time-frame needs. Therefore, they may show different growth and metastatic rates. An orthotopic mouse model often evaluates the endpoint by analyzing tumour volume and survival over a short time-point [218]. In human trials, evaluation of endpoints is complex and depends on age, diet and prior treatment of the patients. In vivo experiments utilizing immunodeficient animals lacks immune cell tumour interaction, while this factor is present in clinical trials [218,219].

Immunotherapy has shown outstanding results in generating T-cell responses and sensitizing the immune system against the tumour cells. More efforts are needed to develop personalized neoantigen vaccination. Patient’s normal and tumour cells can be subjected to RNA and exome sequencing [220]. Then, major histocompatibility complex modelling can be used to rank specific neoantigens expressed by tumour cells. Subsequently, a patient-specific neoantigen-presenting vaccine can be developed to sensitize immune cells against tumour cells.

Efforts should be made to engineer alternative strategies with minimal acute and chronic toxicity to the neurocognitive function. If a drug molecule is penetrating the BBB but in the long term it is damaging healthy cells, the patient’s survival rate increases, but it may affect their quality-of-life. Combination therapy employing novel drug delivery systems and immunotherapy or oncolytic viruses might offer promising results in the near future. Stakeholders may consider enhancing research funding and promoting clinical trials that could assist in evaluating the efficacy of novel strategies. Engineered combinational therapy holds great promise to address the current pitfalls associated with GB treatment.

### 6.1. Drug Delivery as a Biological Decision-Making Process

Traditional drug delivery often treats the tumour as a passive target, but emerging strategies reframe it as an active decision network. Delivery systems can be designed to interact with cellular signalling and “decisions”—for instance, logic-gated nanocarriers that release drugs only when multiple tumour-specific conditions are met [221]. Such systems essentially control when and where a drug is released in the biological milieu, ensuring therapy is activated only in the right context. Researchers developed a nanoparticle that remains inert until encountering dual inputs (e.g., acidic pH and hypoxia), triggering a CRISPR gene-editing payload or drug release [222]. For example, an ROS-dependent pH-responsive nanomedicine system elucidated promising anti-tumour efficacy. Herein, polymersomes were synthesized using multi-responsive amphiphilic block copolymers and hydrophobic blocks. Polymersomes delivered Dox only under the influence of ROS and an acidic pH that is found in solid tumours [223]. Similarly, another study synthesized nano-sized micelles using PEG-block-polycarbonate and performed chemical modifications to achieve the ROS- and pH-responsive delivery of Dox [224].

Similarly, synthetic biology-inspired approaches use AND-gate mechanisms to increase specificity. For example, Researchers engineered antibody pairs that only unleash immune-killing when two antigens are co-recognized on GB cells, acting like a biological and gate for selectivity [225]. These technologies show how drug delivery can transcend passive distribution—instead orchestrating dynamic cell responses. By programming delivery vehicles to sense and respond to intracellular cues or microenvironment signals, researchers are turning therapy into an interactive decision-making process rather than a one-way drug dump, potentially increasing precision and reducing off-target effects [221].

### 6.2. Integrating Tumour Stress Responses into Delivery Design

Glioblastoma’s aggressive resilience arises in part from its stress-adaptive pathways—mechanisms like autophagy, ferroptosis resistance, and T-cell exhaustion help tumours survive therapy. Cutting-edge delivery strategies now aim to co-opt or counteract these responses. For example, autophagy is a pro-survival response to therapy-induced stress that can undermine drug efficacy. Ruan et al. tackled this by co-delivering Dox with hydroxychloroquine (an autophagy inhibitor) in a legumain-activated nanoparticle, while also combining it with anti-PD-L1 immunotherapy [226]. This multiplexed approach blocked the GB cells’ autophagy-based drug escape and immune evasion, yielding enhanced tumour cell-killing in vivo. Similarly, ferroptosis—iron-dependent oxidative cell death—is a vulnerability in GB that can be therapeutically exploited. Cao et al. designed biomimetic macrophage-cloaked nanoparticles that induce ferroptosis in GB by causing catastrophic mitochondrial damage [227]. This triggered the cell death pathway, overcoming certain drug resistances, and can synergize with the immune attack. Notably, stress pathways and immunity intersect: Wang et al. showed that CD8^+^ T-cells drive ferroptosis in cancer cells during immunotherapy [228], found at https://pmc.ncbi.nlm.nih.gov/ (accessed on 24 December 2025), linking immune-induced oxidative stress to tumour control. Incorporating such insights—e.g., packing ferroptosis inducers, autophagy blockers, or agents to reverse T-cell exhaustion—into delivery systems turns tumour stress responses against the tumour, improving therapeutic outcomes.

### 6.3. System-Level and Adaptive Design Principles in GB Therapy

To surmount GB’s complexity, researchers are adopting system-level design principles—viewing the tumour and treatment as an integrated, adaptive system. This includes leveraging big data and machine learning to inform nanoparticle design. In a recent study, Mendes et al. assembled data from 745 cancer nanomedicine studies and used explainable machine learning to identify design rules: for instance, nanoparticle shape and therapy modality emerged as key determinants of in vivo efficacy [229]. Such analyses guide more-holistic optimization of delivery vehicles (beyond trial-and-error), ensuring they are tuned to the tumour’s system biology. Another emerging principle is real-time adaptivity. Therapies can be engineered to respond to the evolving tumour microenvironment or external controls. For example, Wu et al. created CAR T-cells with a heat-sensitive switch, so that focused ultrasound triggers local CAR T activation on demand [230]. This kind of closed-loop control—using an external stimulus or an internal biosensor feedback—exemplifies adaptive delivery, maximizing on-tumour effect while sparing healthy tissue. Similarly, delivery platforms are being developed with built-in sensors (pH, enzymes, etc.) that continuously sense and adjust drug release in response to tumour signals. By integrating system biology, computational modelling, and adaptive feedback mechanisms, next-generation GB drug delivery systems aim to dynamically sharpen treatment responses amidst heterogeneity and changing tumour states [229].

## 7. Conclusions

Despite remarkable technological progress, glioblastoma remains one of the most treatment-refractory human malignancies. As comprehensively reviewed here, a wide spectrum of drug delivery platforms—including nanoparticles, hydrogels, extracellular vesicles, microneedles, implantable wafers, and convection-enhanced delivery systems—have been developed to overcome the blood–brain barrier and improve intra-tumoural drug accumulation. Yet, the limited clinical translation of these approaches reveals a fundamental disconnect between delivery engineering and tumour biology. Most strategies successfully answer the question of how to deliver drugs, but insufficiently address how tumour cells interpret and respond to sustained or localized therapeutic stress.

A key reason many delivery strategies fail is that enhanced drug exposure alone does not guarantee tumour cell elimination. Prolonged or spatially confined drug release often induces adaptive stress responses, including autophagy activation, metabolic reprogramming, DNA damage tolerance, and immune escape, which collectively enable GB cells to survive otherwise lethal insults. In this context, delivery systems designed to maximize residence time may inadvertently stabilize cytoprotective programmes rather than drive irreversible cell death. Moreover, passive targeting approaches based on static features—such as receptor overexpression or enhanced permeability—do not account for the dynamic remodelling of the GB microenvironment, including fluctuating hypoxia, vascular heterogeneity, immune infiltration, and extracellular matrix stiffness. As GB evolves, these changes reshape intracellular trafficking, signalling hierarchies, and therapeutic sensitivity, rendering many initially effective delivery systems progressively ineffective.

Addressing these limitations requires a conceptual shift in how drug delivery is designed and evaluated. Rather than viewing delivery platforms as inert carriers, future strategies must treat them as biological instructors capable of actively shaping tumour cell fate. This entails designing delivery systems that deliberately engage or suppress specific stress-response pathways—such as autophagy, ferroptosis, and immune exhaustion—to bias tumour cells toward apoptotic, ferroptotic, or immunogenic outcomes. Importantly, biological endpoints such as autophagic flux, lipid peroxidation, immune activation, and checkpoint signalling should be incorporated as core design criteria alongside pharmacokinetics and biodistribution.

Equally critical is the adoption of system-level and adaptive design principles. Machine learning, network biology, and integrative modelling offer powerful tools to decode complex relationships between delivery parameters and biological outcomes, enabling the rational prioritization of delivery strategies before extensive in vivo testing. Adaptive or logic-gated platforms that respond to tumour-specific cues—such as redox state, enzymatic activity, or inflammatory signals—represent a promising direction to accommodate intra-tumoural heterogeneity and tumour evolution.

In summary, the future of GB drug delivery lies in moving beyond static engineering solutions toward biologically informed, dynamic, and outcome-driven systems. Aligning delivery logic with tumour decision-making programmes will be essential to transform drug delivery from an enabling technology into a decisive therapeutic strategy against glioblastoma.

## Figures and Tables

**Table 1:** Different nanoparticle-based drug delivery systems used for GB treatment.

Nanoparticle	Drug	Type	Observations	References
Fe_3_O_4_	Ferumoxytol	Metal oxide	Ionizing radiation degrades Ferumoxytol core to release Fe^2+^ ions to enhance H_2_O_2_-dependent GB cell killing	[91]
TiO_2_–Pt	Platinum	Metal oxide	Reduce tumour growth	[92]
1,2-Dioleoyl-sn-glycero-3-phosphorylcholine, 1,2-dioleoyl-sn-glycero-3-phosphoethanolamine	Ursolic acid and Dox	Liposome	Dox-induced cardiotoxicity is lowered Drug delivery platforms prolong median survival of GB-bearing mice	[93]
MnO_2_	Ce6	Metal oxide	Macrophage-coated nanoparticles activate immature dendritic cells and cGAS (cyclic GMP-AMP synthase)-STING (stimulator of interferon genes) pathway Enhances ROS production	[94]
Silica nanoparticles	Paclitaxel	Metal	An acidic environment triggers drug release to induce cytotoxicity in U87 cells	[95]
Graphitic carbon nitride, (g-C_3_N_4_)	g-C_3_N_4_	Polymer	Nanoparticles arrested the cell cycle at the S phase in LN229 and SNB19 GB cells	[96]
Phosphatidylcholine, lipid medium-chain triglycerides, solid lipid the myristyl myristate	Chloro-aluminum phthalocyanine	Lipid	630 nm light irradiation induced ROS production and reduced brain tumour cell viability and proliferation	[97]
1,2-distearoyl-sn-glycero-3-phosphoethanolamine-N-[amino(polyethylene glycol)-2000] (DSPE-PEG2000),	TMZ, miR603 and miR221	Liposomes	Formulations lowered the tumour size and enhanced the median survival of mice	[98]
CpG and polyinosinic/polycytidylic	Dox, CpG and polyinosinic/polycytidylic	Liposomes	Liposome treatment activates dendritic cells and macrophages	[99]
DODMA/DOPE,	miR-181a	Lipid	Induced cellular death of U87 GB cells Delayed tumour growth in an in vivo tumour model	[100]
Chitosan-PLGA	Gemcitabine	Polymeric	Improved cytotoxicity observed in human GB cells	[101]
HA-diketopyrrolopyrrole		Polymeric	Nano-formulations effectively targeted CD44+ GB cells	[102]
PEG-PLGA	Paclitaxel and etoposide	Polymeric	In vitro study showed NPs have lower IC_50_ values compared to pure drugs	[103]
PEG-PLGA	Bevacizumab and dichloroacetate	Polymeric	CD147-targeting peptide surface functionalization enhanced drug retention in tumour cells	[104]

**Table 3:** Clinical trial records of different strategies to treat glioblastoma.

Treatment Arm	Phase (No of Enrolments)	Outcome	Clinical Trial Identifier	Reference
Pembrolizumab	I (110)	Ongoing	NCT02359565	
Neoadjuvant nivolumab and adjuvant nivolumab	I (45)	Ongoing	NCT04323046	
IMA950	I (45)	IMA950 plus GM-CSF was well-tolerated	NCT01222221	[200]
Epidermal growth factor receptor variant III peptide vaccination	II (40)	Median overall survival—26 months	NCT00643097	[201]
APVAC1 and 2 vaccine plus polyICLC and GMCSF concurrent to TMZ	I (16)	Overall survival—29 months Progression-free survival—15.2 months	NCT02149225	[202]
ICT-107	II (124)	Overall survival—38.4 months Progression-free survival—16.9 months	NCT01280552	[203]
Recombinant poliovirus	I (61)	The survival rate among patients who received this therapy was higher at 24 and 36 months than the rate among historical controls	NCT01491893	[204]
Antigen-expressing measles virus (MV-CEA)	I (23)	Overall survival—11.6 months	NCT00390299	[205]
Human epidermal growth factor receptor 2 (HER2-specific chimeric antigen receptor (CAR)-modified virus-specific T-cells	I (16)	Overall survival—11.1 months	NCT01109095	[206]
Pegylated liposomal doxorubicine	I/II (63)	Overall survival—17.6 months	NCT00944801	[207]
RNA–lipid particle (RNA-LP) vaccines	I (24 estimated)	Recruiting	NCT06389591	
Rhenium-186 nanoliposomes	I (40 estimated)	Recruiting	NCT05460507	
RNA–lipid particle (RNA-LP)	I (28 estimated)	Recruiting	NCT04573140	
Liposomal curcumin	I/II (30)	Recruiting	NCT05768919	
Panzem nanocrystal colloidal dispersion	II (15)	Completed	NCT00481455	
Autologous dendritic cell vaccination (ADCV01)	II (24 estimated)	Recruiting	NCT04115761	
AZD1775	0 (20)	Completed	NCT02207010	
Allogeneic γδ T-cells (genetically edited with ARIH1 and BCL11b knockout)	Not applicable (18)	Recruiting	NCT07144735	
Mibefradil with radiation	I (18)	Dose-limiting toxicities were observed	NCT02202993	[208]
DOC1021 Dendritic cell immunotherapy	II (180 estimated)	Recruiting	NCT06805305	
BEY1107 + TMZ	I (12 estimated)	Recruiting	NCT05769660	

## Data Availability

No new data were created or analyzed in this study. Data sharing is not applicable to this article.

## Abbreviations

Akt	Protein kinase B
ANG-1/2	Angiotensin I or angiotensin II
BBB	Blood–brain barrier
BEV	Bevacizumab
BSA	Bovine serum albumin
BTB	Blood–tumour barrier
CD2AP	CD2-associated protein
CD73	Cluster of differentiation 73
CDA	Cytosine deaminase
CDKN2A	Cyclin-dependent kinase inhibitor 2A
CNS	Central nervous System
–CONH	Amide functional group
COOH	Carboxyl group
CpG	Dinucleotide
DCs	Dendritic cells
DNA	Deoxyribonucleic acid
Dox	Doxorubicin
ECM	Extracellular matrix
EGFR	Epidermal growth factor receptor
EMP1	Epithelial membrane protein 1
EMT	Epithelial–mesenchymal transition
EPR	Enhanced permeability and retention
FDA	US Food and Drug Administration
FGF	Fibroblast growth factors
GB	Glioblastoma
GPCRs	G-protein-coupled receptors
GSCs	Glioblastoma stem-like cells
GTP	Guanosine triphosphate
HA	Hyaluronic acid
HIF-1	Hypoxia-inducible factor 1
HIV-AIDS	Human immunodeficiency virus infection and acquired immune deficiency syndrome
IDH1	Isocitrate dehydrogenase gene 1
IFN-γ	Interferon-gamma
IGFBP4	Insulin-like growth factor binding protein 4
IĸBα	Inhibitor of nuclear factor kappa-B alpha
IL-6	Interleukin-6
IL-8	Interleukin-8
KIFs	Kinesin superfamily proteins
KLF4	Krüppel-like factor 4
LAG-3	Lymphocyte-activation gene 3
LRP	Lipoprotein receptor-related protein
MAPK	Mitogen-activated protein kinase
MDSCs	Myeloid-derived suppressor cells
MGMT	O6-methylguanine-DNA methyltransferase
MnO_2_	Manganese dioxide
mRNA	Messenger RNA
MSCs	Mesenchymal stem cells
mTOR	Mammalian target of rapamycin
NEDD41	E3 ubiquitin-protein ligase
NF-ĸβ	Nuclear factor kappa-light-chain-enhancer of activated B cells
NH_2_	Amino functional group
NSCs	Neural stem cells
OCT4	Octamer transcription factor 4
OH	Hydroxyl group
p50	NF-κB subunit
p65	NF-κB subunit
PAMAM	Poly(amidoamine)
PCL	Polycaprolactone
PDGFA	Platelet-derived growth factor subunit A
PD-L1	Programmed death-ligand 1
PEG	Poly ethylene glycol
PI3K	Phosphoinositide 3-kinase
PLA	Polylactic acid
PLGA	poly(lactic-co-glycolic) acid
PVA	Poly vinyl alcohol
PVP	Polyvinylpyrrolidone
RAF	Rapidly accelerated fibrosarcoma (serine/threonine-specific protein kinases)
RAFT	Reversible addition–fragmentation chain transfer
RNA	Ribonucleic acid
ROS	Reactive oxygen species
SO_3_H	Sulfonic acid
TGF-β	Transforming growth factor beta
TIM-3	T-cell immunoglobulin and mucin domain 3
TMEM17	Transmembrane protein 17
TMZ	Temozolomide
TNF-α	Tumour necrosis factor alpha
TSPAN4	Tetraspanin 4
VEGFA	Vascular endothelial growth factor
